# Morphometics and Gonadal Development of the Hagfish *Eptatretus cirrhatus* in New Zealand

**DOI:** 10.1371/journal.pone.0078740

**Published:** 2013-11-08

**Authors:** Frederic H. Martini, Alfred Beulig

**Affiliations:** 1 Department of Oceanography, University of Hawaii at Manoa, Honolulu, Hawaii, United States of America; 2 Division of Natural Sciences, New College of Florida, Sarasota, Florida, United States of America; National Institute for Basic Biology, Japan

## Abstract

Hagfishes have been the target of commercial fisheries in many areas of the world, with the catch processed for leather and for human consumption. A fishery has been operating in New Zealand waters for the last six years, harvesting the bearded hagfish, *Eptatretus cirrhatus*. The fishery has thus far been unregulated. Based on samples collected dockside over a two-year period, this report expands the morphometric database for this species, provides information on the size and weight of the harvested animals, determines the sizes at the onset of gonadal development and the minimum sizes at sexual maturation for males and females, and indicates that *E. cirrhatus*, like most other hagfish species, has no specific breeding season. Although females appear in the population at smaller sizes, the sex ratio for mature animals is 1:1 and the sizes of the largest males and females are comparable. The changes observed in sex ratio as a function of TL suggest differences in the timing and rates of gonadal development in females versus males rather than protogyny. Based on the size of the eggs, the number of eggs per female, the proportion of the population that contains large eggs, and the number of postovulatory females, it is clear that *E. cirrhatus*, like other hagfish species, are potentially vulnerable to overexploitation.

## Introduction

The hagfishes, or Myxinoidea, are worldwide in distribution, with 79 species described and at least 4 more awaiting formal description. All known species live in close association with the bottom, resting on the substrate or occupying burrows within soft sediments [1,2]. Hagfish populations can be very dense and have large energetic demands [3,4] that are collectively too high to be sustained by carrion scavenging alone. Caloric requirements may be met by predation [5], supplemented by opportunistic scavenging [6], chemoautotrophic production [7,8,9], and nutrient absorption across the epithelia of the gills and skin [10]. 

Several species of hagfish have significant commercial value. The skins are used for “eelskin” leather products and the meat is considered a delicacy in Korea and some regions of Japan [11]. *Eptatretus cirrhatus*, the bearded hagfish, is relatively common in the coastal waters of New Zealand and southern Australia. For the last six years, *E. cirrhatus* populations in New Zealand have been the target of a small-scale commercial fishery supplying the Korean market with both fresh and frozen product. Over this period, the largest vessel was the 72 m F/V ShinJi, a Korean-flag vessel working under charter to Tuere Fisheries, Limited, of Christchurch. Although NZ Fisheries officers sometimes visited the ShinJi while it was offloading, landings were not closely monitored and this NZ fishery was unregulated. 

Little is known about the biology of *E. cirrhatus*, and the anatomical descriptions are based on a relatively small number of specimens that have been examined by different investigators at different times using different reporting methods. The goals of this study were (a) to expand the morphological data-set and (b) to obtain information about the reproductive biology of this species of potential value to fishery management. 

## Materials and Methods

The hagfishing vessel ShinJi (Figure S1A) worked within 100 nautical miles of the eastern and western shores of New Zealand year-round, always remaining outside of the 12 mile territorial limit. Strings of 200 L plastic barrels with entrance funnels and 17 mm escape holes (Figure S1B) were baited with fish waste or commercial bait and set on the bottom at 400-500 m for 1-2 days at a time. On retrieval, the catch was sorted and packaged, then frozen and stowed in the freezer hold. The ship remained at sea for 3-6 weeks at a time. When the freezer space was filled, live hagfish were placed in a capacious live-well aerated by compressors, and when the live-well reached capacity the ship headed to the nearest commercial port, and both fresh and frozen product offloaded and shipped to Korea for processing. 

We obtained specimens from this ship at roughly 3 month intervals from October 2009 through June 2011, which corresponded to the times the ship was offloading in the ports of Auckland or Onehunga. When possible, a variable number of freshly dead specimens were collected from the holding tank onboard and 1-2 prepackaged frozen blocks of hagfish (Figure S1C, D), each block weighing approximately 25 kg (60 lb), were selected at random from the conveyor belt offloading the catch. Because of the sampling method, the precise origin of each sample could not be determined, and the company declined requests to release related information on the fishery operation, such as total catch, days at sea, cumulative landings, or discard rates. 

A total of 393 specimens were collected from the ship through visits in October 2009 (*n* = 132), February 2010 (*n* = 69), July 2010 (*n* = 126), October 2010 (*n* = 51) and June 2011 (*n* = 15). Individuals with unusual color variations, tail shapes, injuries, or scars were photographed with a Nikon D-3000 digital camera. All specimens were measured, weighed, and examined for reproductive state. No permits were required for specimen collection from the commercially licensed vessel, which complied with relevant fisheries and governmental regulations. Permits were obtained by the lead investigator to handle formalin and to obtain 95% ETOH for specimen and tissue storage, respectively. 

Detailed morphological information was obtained from 178 fresh specimens. Methods of measuring and counting followed those of Fernholm and Hubbs [12] and McMillan and Wisner [13]. The body axis was divided into 4 regions (prebranchial, branchial, trunk, and tail). The sum of these measurements is equal to the total length (TL). 

Trunk depth is the maximum, exclusive of fin fold, measured at the midpoint of the trunk. Cloacal depth is measured at the anterior portion of the cloaca, excluding the dorsal fin fold, whereas tail depth is the perpendicular depth of the entire tail, including the dorsal and ventral fin folds. Cusp counts (unicusps and multicusps on outer and inner rows) were recorded for both sides to obtain the total cusp count. When counting slime pores, the same regional definitions were used as with the length data. All measurements were taken from the left side of the body, and recorded in millimeters. Gill openings were counted on each side, but gill pouches were counted only in the rare instances when there were more than 7 gill openings on one side. Measurements were taken from a fish-board, depths were determined by caliper, and weights in grams were taken from a digital scale. With regard to analyzing morphometric/meristic parameters such as length/weight relationships or body part counts, we fitted curves to the data and compared the goodness of fit of linear versus non-linear regression equations. Determination of significance of the relationships among parameters as descriptors of body proportions as well as comparisons of these measures among seasons, between sexes, or among size classes were then made in relation to total length (TL) using the F-test. We also carried out correlational analyses (Pearson) with meristic characters. We used the standard fisheries equation (W= aL^b^ , where W= weight, L= length, a= a coefficient and b=an exponent) to depict length-weight relationships in assessing and comparing condition. Because it was necessary to freeze some specimens for later analysis, we were also interested in whether the process of freezing caused differences in weight and/or length between fresh and formerly frozen material in a non-linear manner. Accordingly, we fitted fresh and frozen values in length/weight (L:W) to second order polynomials and compared curve parameters with the F-test. 

Because representative specimens collected in this study would be preserved and retained, 24 specimens were weighed, measured, and preserved in 10% formalin in seawater for 6 months, then weighed and measured a second time, to assess the impact of preservation on both length and weight. We compared the weights and lengths before and after treatment with the matched t-test as there were too few data points for a meaningful non-linear analysis of weight and length together. 

The gonads in hagfishes develop within a mesenterial fold located to the right of the dorsal mesentery that supports the gut. The anterior two-thirds of the gonad may develop into ovarian tissue, and the posterior one-third may develop into testicular tissue. Reproductive state as reported here was determined by opening the entire length of the peritoneal cavity and inspecting the mesentery using a hand lens or dissecting scope; histological materials from each stage were preserved for later analysis. If present, developing eggs were measured and the maximum egg dimensions recorded using calipers. When large eggs or postovulatory follicles were present, they were both measured and counted. 

**Figure 1:**
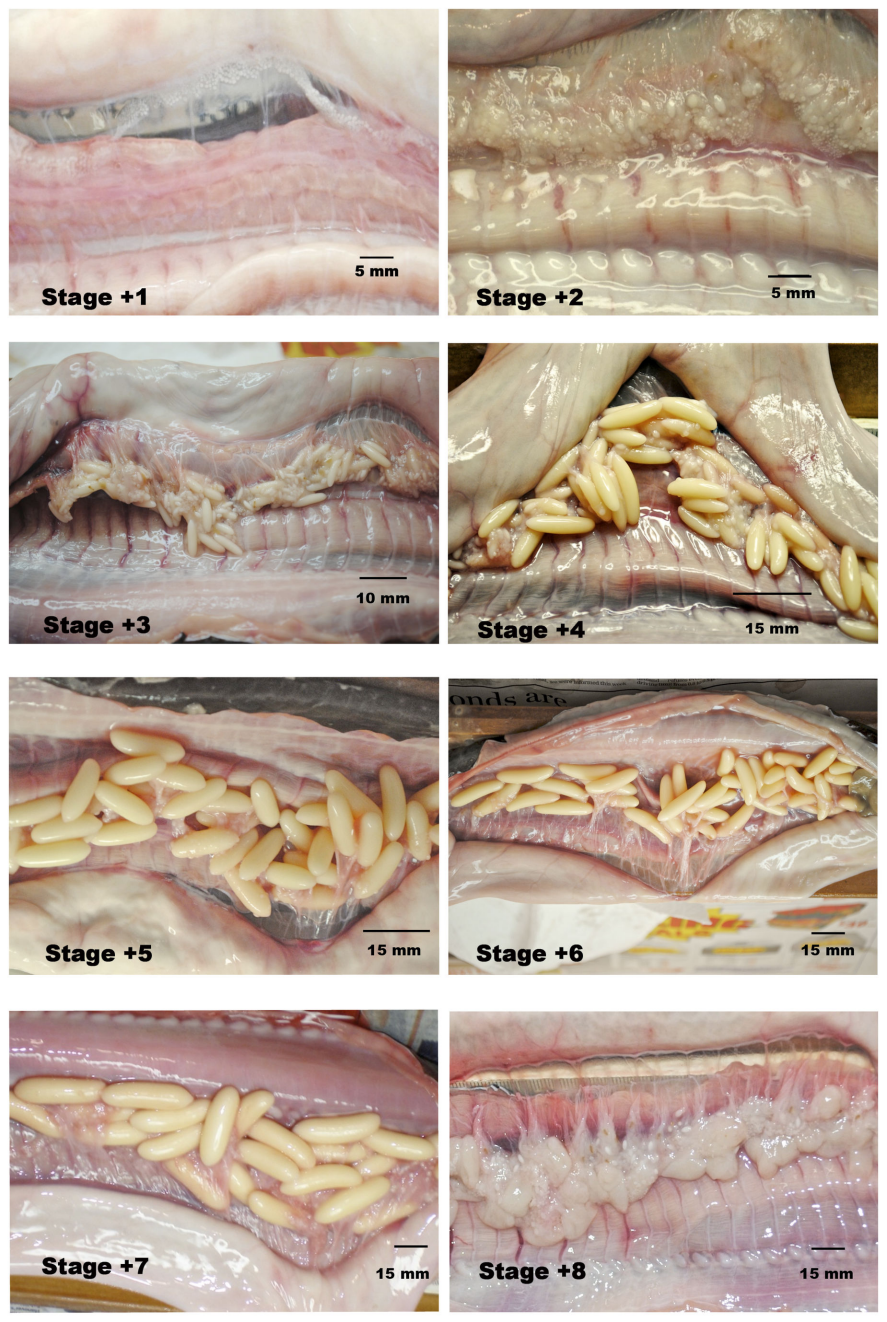
Stages of female sexual maturation (see Table 1).

**Figure 2:**
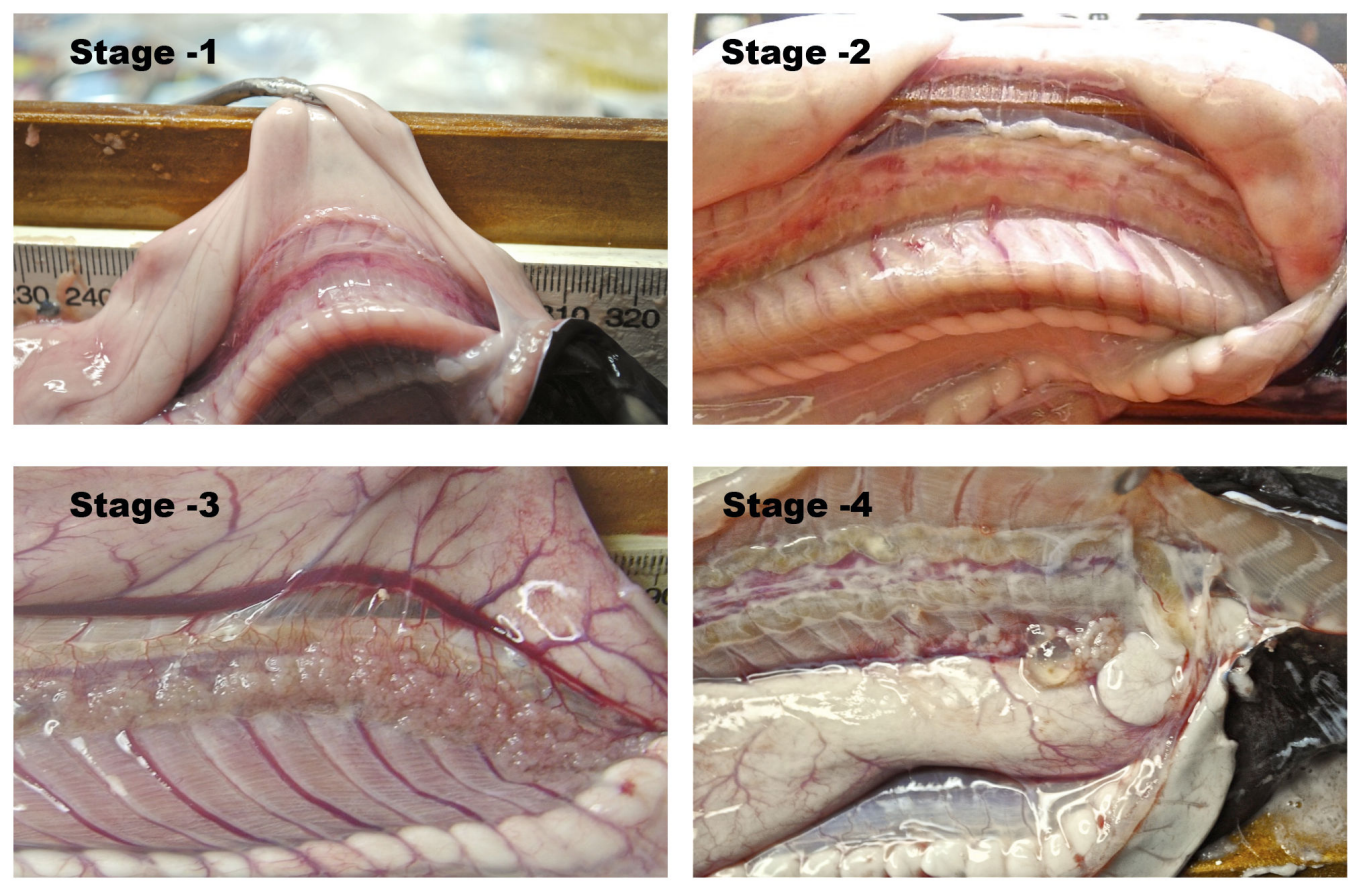
Stages of male sexual maturation (see Table 1).

The samples contained a mixture of immature, undifferentiated animals and sexually differentiated individuals. Each specimen was assigned to a reproductive stage based on testicular follicle structure and cloacal gland size (males) or ovarian follicle length or condition (females). These stages, detailed in Table 1 and Figures 1-2, are similar to those used in an earlier investigation of *Myxine glutinosa* [4]. Staging was verified by histological examination of representative members of each stage. 

**Table 1:** Stages of Sexual Differentiation.

**Classification**	**Description**
Stage 0	Indeterminate or undifferentiated; no identifiable gonadal development
Females (See Figure 1):	
Stage +1	largest eggs <1 mm
Stage +2	largest eggs 1-3 mm
Stage +3	largest eggs 4-7 mm
Stage +4	largest eggs 8-15 mm
Stage +5	largest eggs 16-23 mm
Stage +6	largest eggs 24-31 mm
Stage +7	largest eggs 32-36 mm
Stage +8	large, flaccid postovulatory follicles indicating recent spawning activity
Males (See Figure 2):	
Stage -1	testicular band present
Stage -2	small testicular follicles containing fluid
Stage -3	enlarged testicular follicles, cloacal gland <12 mm in diameter
Stage -4	distended testicular follicles, cloacal gland 12-17 mm in length

When looking for trends in reproductive activity, samples were either grouped by season [Spring = late October, Summer = early February, Fall = late May, and Winter = early August] or sorted into subgroups by TL (50 mm length increments). We compared L/W curves and reproductive stage distributions among seasons with non-linear regressions and the F-test. Weights were transformed into logarithms to reduce the spread of scores and facilitate comparisons among data sets of different *n*-values. All statistical data were analyzed using Prism™ software, v.5 (GraphPad Software, La Jolla, CA).

## Results

### General morphology

**Figure 3:**
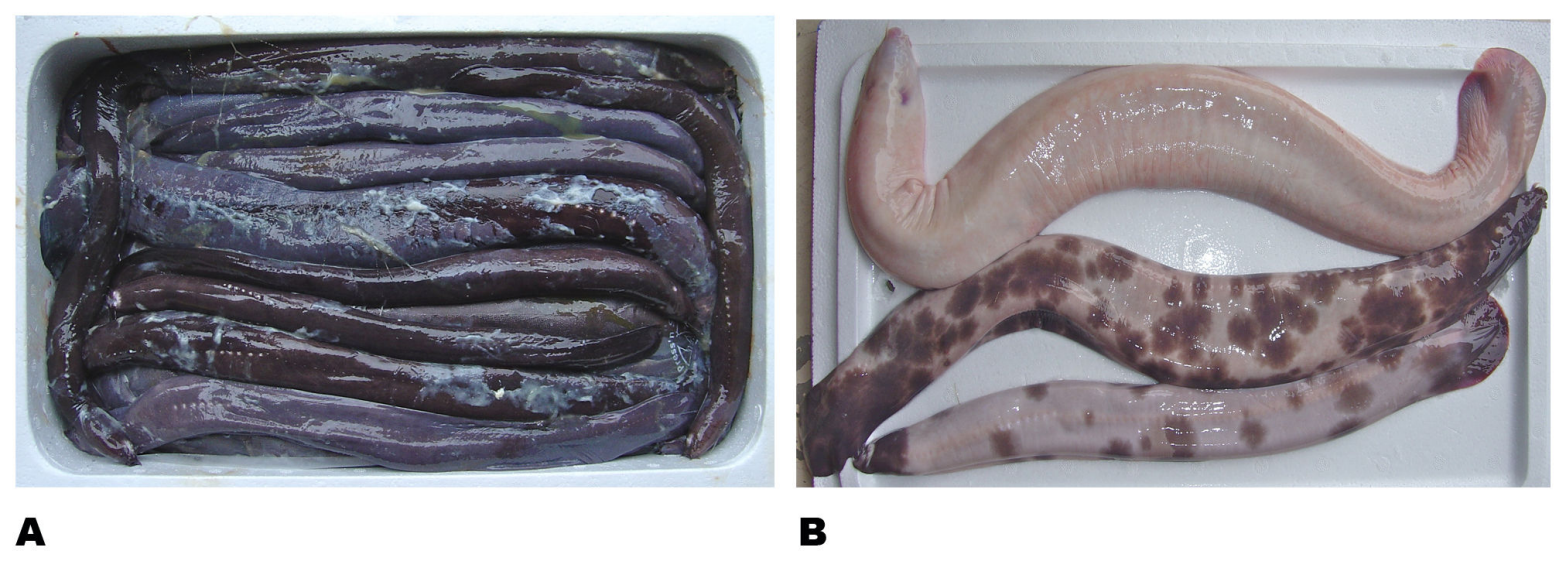
Color variations in *Eptatretus cirrhatus.* (a) Typical fresh specimens have a solid color ranging from burgundy to a chocolate brown. (b) These are two of three piebald specimens and the only albino animal collected.

As with many other hagfish species, the color of *E. cirrhatus* is generally unremarkable. Small animals (<500 mm) are usually a dark blue/purple gray. The eyespots are pale white, as are the openings of the branchial efferent ducts and sometimes the adjacent slime pore ducts. Larger animals may be similarly colored, but they may have other color patterns; it is difficult to determine if these patterns only developed later in life or if they were present earlier but missed due to their rarity. The most common variation is a medium chocolate brown color, followed by a dark burgundy (Figure 3A). Either color form may contain a pale mottling that sometimes merges to form pale blotches. Rarer still is a piebald coloration; the rarest color form is an albino state, seen only once in this study (Figure 3B). Comparable blotchy, mottled, piebald, and albino color variations were reported for *Eptatretus burgeri* [14] and seen in a feeding swarm of *E. deani* (Martini, unpublished observation). 

In addition to these color variations, most animals were marked by small circular white scars from 2-4 mm in diameter. These scars, which heal to pale spots that may ultimately heal completely, mark the attachment sites of large (to 30 mm TL) monogenean ectoparasites, probably Lophocotyle novazeelandica [15] that are the focus of a separate study. 

**Figure 4:**
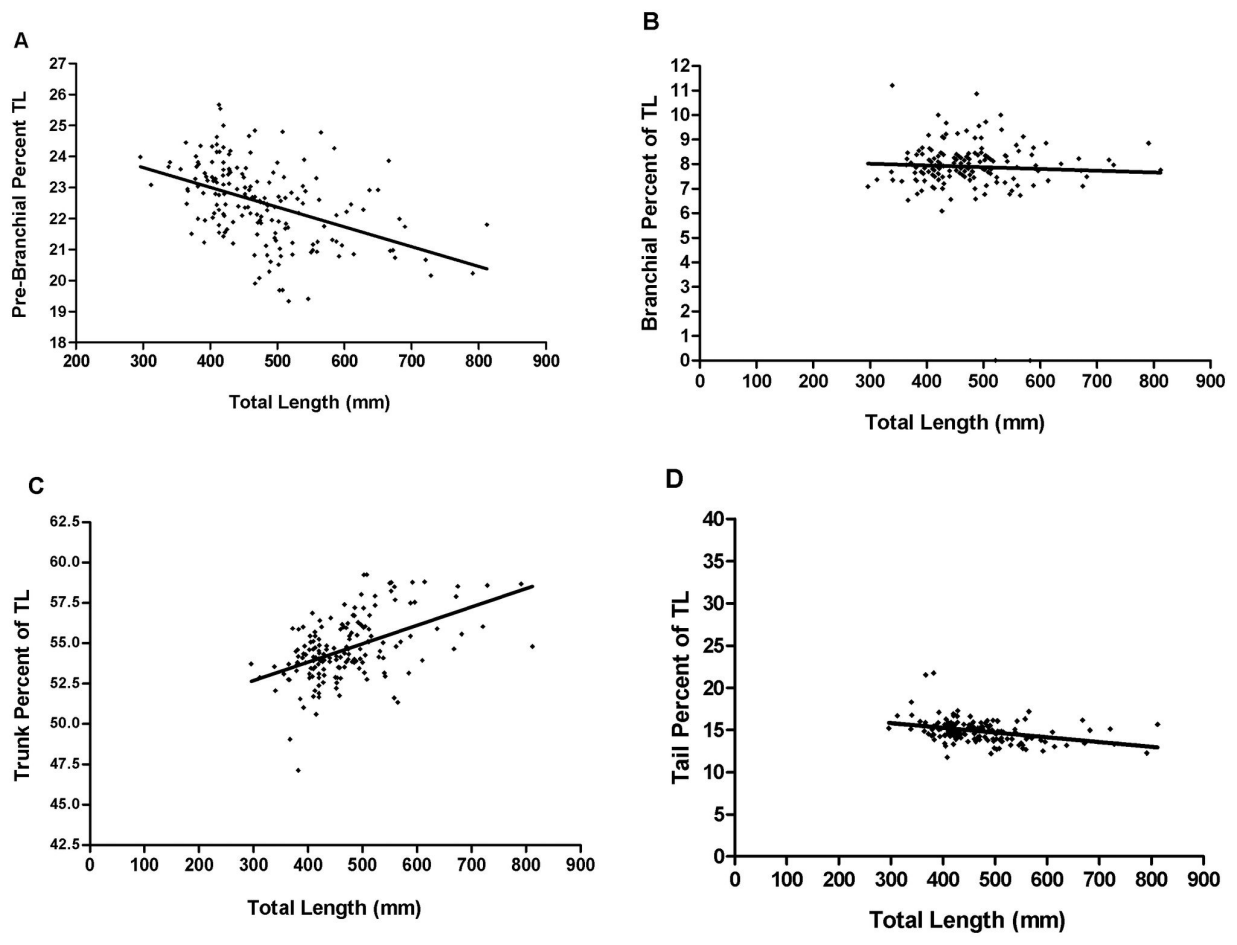
Growth and changes in proportional measurements. (a) Prebranchial length, as percent of TL, plotted against TL. As the hagfish grow, the relative size of the prebranchial region decreases significantly. [Slope = -0.0069; r^2^=0.207; Sy.x=1.218; F_(1,176)_= 46.01; p<0.05*, significant]. (b) Branchial length, as percent of TL, plotted against TL. The relative size of the branchial region does not change with growth. [Slope = -0.0007; r^2^=0.003; Sx.y=1.142; F_(1,170)_=0.484; p>0.05, not significant]. (c) Trunk length, as percent of TL, plotted against TL. As the hagfish grow, the relative size of the trunk increases significantly. [Slope = 0.0082; r^2^=0.063; Sy.x=2.692; F_(1,170)_=11.27; p<0.05*, significant]. (d) Tail length, as percent of TL, plotted against TL. As the hagfish grow, the relative size of the tail decreases significantly. [Slope= -0.0048; r^2^=0.039; Sy.x=2.011; F_(1,170)_=6.892; p<0.05*, significant].

Table 2 summarizes the detailed morphometric data for the *Eptatretus cirrhatus* in this study (n = 393), which had a mean length of 486 mm and a median length of 468 mm. Other morphometric values (*n* = 178) were approximately normally distributed without discontinuities or bimodality that could signify the presence of sympatric species. When the prebranchial length was expressed as a percentage of TL, the nonlinear regression line had a significant negative slope [F_(1,176)_ =46.01; p<0.05: Figure 4A]. This was also true of tail length [F_(1,170)_=6.892; p=<0.05: Figure 4D]. The trunk length regression line had a significant positive slope [F_(1,170)_=ll.27; p<0.05: Figure 4C], whereas the regression line for branchial length (Figure 4B) was not significantly different from zero [F_(1,170)_=0.484; p>0.05]. This indicates that as the hagfish grow, the prebranchial and tail regions form a significantly smaller percentage of the total length while the trunk length increases proportionately. 

**Table 2:** Morphological Measurements for *Eptatretus cirrhatus*.

**Character**	**Mean**	**SD**	**Range**	***n***
Total length (mm)	486	109.1	225-812	393
Weight (g)	303	200.9	29-1050	393
Prebranchial Length (%TL)	22.6	1.4	19.7-24.8	178
Branchial Length (%TL)	7.6	1.8	6.6-11.2	178
Trunk Length (%TL)	52	11.6	44.1-59.3	178
Tail Length (%TL)	14.3	3.7	8.6-21.5	178
Depth, trunk (%TL)	6.4	0.84	5.1-9.2	177
Depth, cloaca (%TL)	5.6	0.71	4.4-7.5	177
Depth, tail (%TL)	8.5	1.04	5.5-10.5	177
Total cusps	48	1.2	44-52	178
Multicusps, outer/inner	3/3			178
Unicusps, outer/inner			8-10/8-10	178
Total slime pores (left side)	82	5.5	74-90	178
Prebranchial slime pores	16	0.9	13-18	178
Branchial slime pores	6.3	0.5	6-7	178
Trunk slime pores	49	1.5	46-54	178
Tail slime pores	11.7	0.9	10-14	178

Although the feeding apparatus, consisting of the tooth cusp plates and the dental muscle complex in the prebranchial region [16], is therefore relatively large in smaller individuals, the number of tooth cusps remains unchanged.

**Figure 5:**
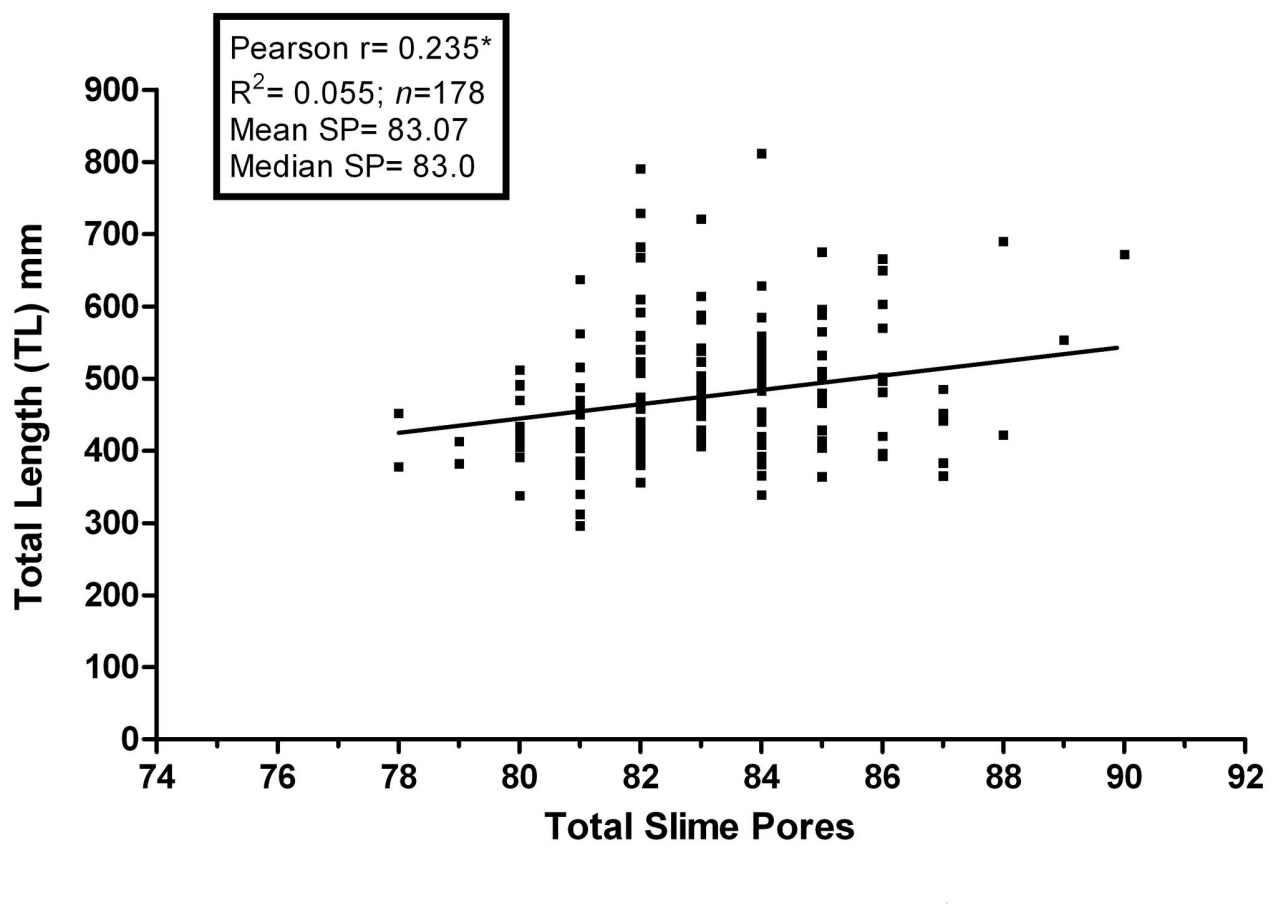
Total slime pore count plotted against TL. The number of slime pores increases significantly as the hagfish grow in length (p<0.05*).

**Figure 6:**
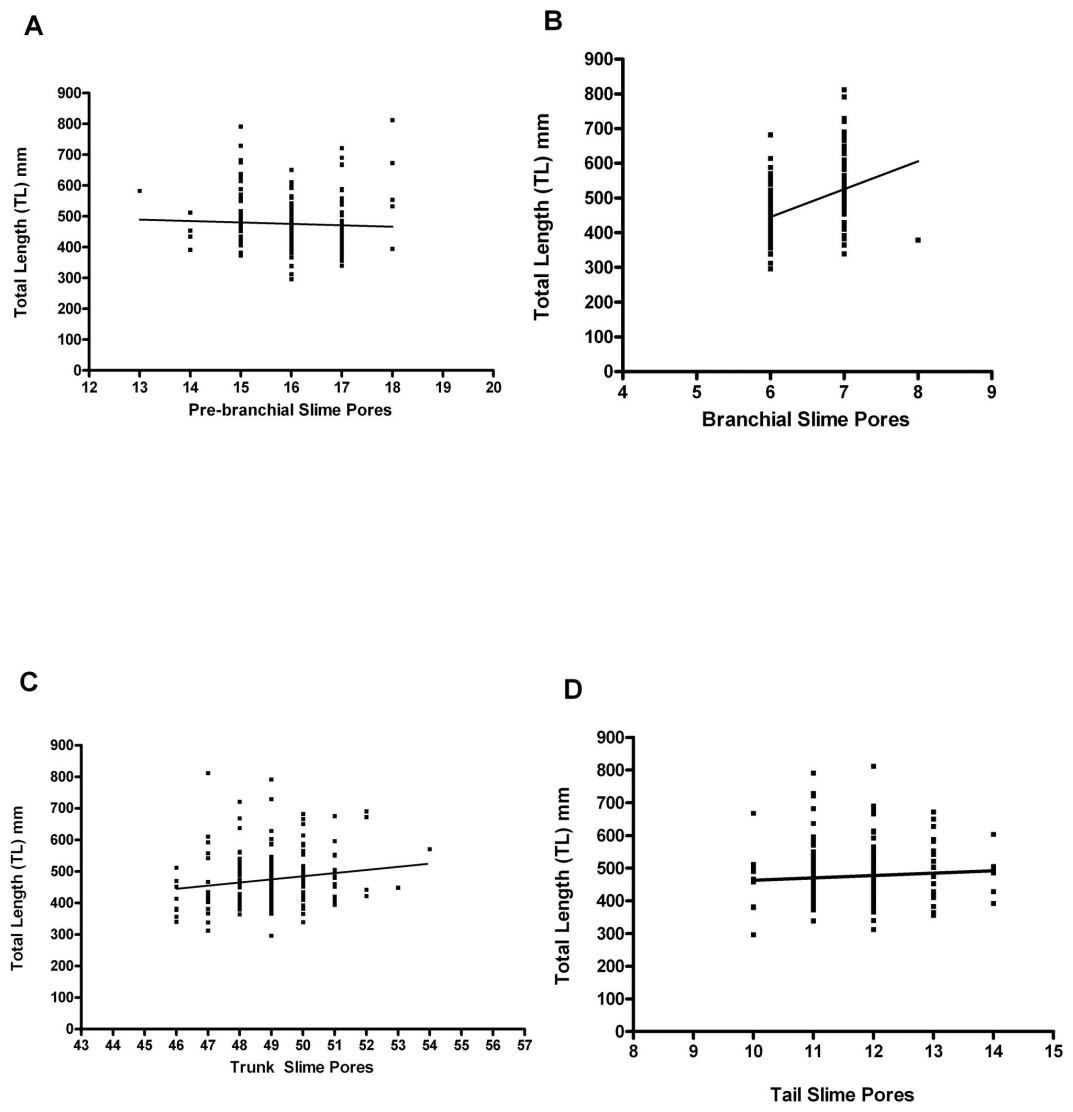
Growth and changes in slime pore number and distribution. (a) Prebranchial slime pore count plotted against TL. The slime pore count in this region does not change significantly with growth. [Pearson r=-0.046; p>0.05, not significant; R^2^=0.002, *n*=178; mean 16.02, median 16]. (b) Branchial slime pore count plotted against TL. The number of slime pores in the branchial region increases significantly with growth, as 7 slime pores become more common. [Pearson r=0.447; p<0.05*, significant; R^2^=0.197; *n*=178; mean 6.37, median 6.0]. (c) Trunk slime pore count plotted against TL. The number of slime pores in the trunk increases with growth. [Pearson r=0.165; p<0.05*, significant; R^2^=0.027; *n*=178; mean 49.0, median 49.0]. (d) Tail slime pore count plotted against TL. The slime pore count in this region does not change significantly with growth. [Pearson r=0.077; p>0.05, not significant; R^2^=0.006; *n*=178; mean 11.69, median 12.0].

The total number of slime pores increases with TL (Pearson r=0.235; p<0.05: Figure 5). However, this pattern is not uniformly distributed over all regions of the body; no increases were found in either the pre-branchial region (Pearson r= -0.046; p>0.05: Figure 6A) or the tail region (Pearson r=0.077; p>0.05: Figure 6D). The overall increase in slime pore count with TL resulted from (1) a tendency for larger hagfish to have seven slime pores in the branchial region rather than six (Pearson r=0.447; p<0.05: Figure 6B) and (2) an increased number of slime pores in the trunk region (Pearson r=0.165; p<0.05: Figure 6C) proportional with increase in the size of the trunk as growth occurs.

In our study, three of the animals examined had eight gill openings on the left side. In two of these animals, the last branchial efferent on the left side had not fused with the pharyngocutaneous duct, and the gill pouch count was 14. However, one animal was found on dissection to have eight gill pouches on the left side, rather than seven; the right side had the usual seven gill pouches, for a total gill pouch count of 15. 

**Figure 7:**
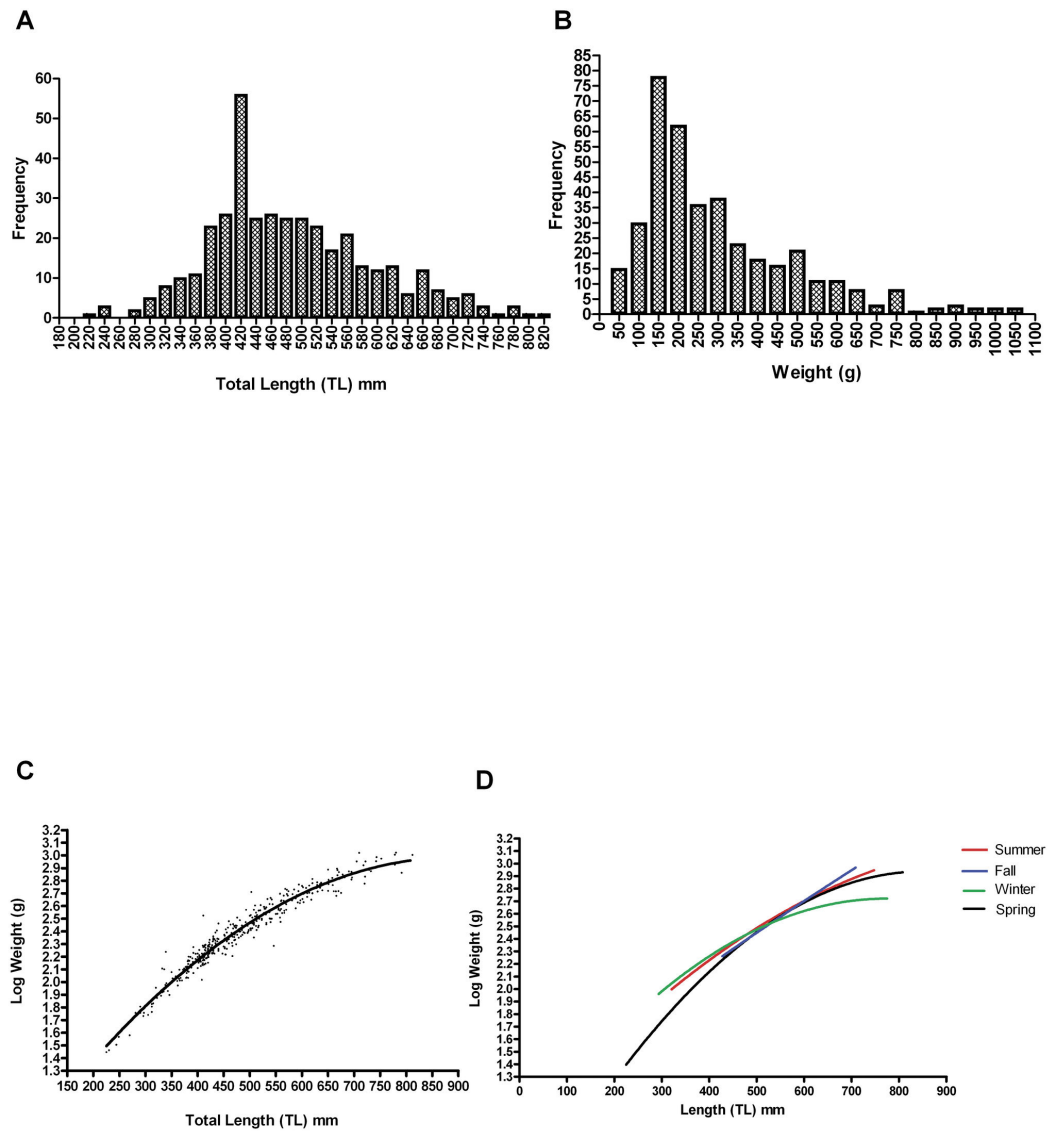
Length and weight data. (a) A histogram of total length (TL) data for this study (see Table 2 for related statistics). (b) A histogram of weight data for this study (see Table 2 for related statistics). (c) A non-linear regression curve of log weight on TL. [LogW=a+bL+cL^2^; a=0.320, b=0.00022, c=-3.347e-006; R^2^=0.951; Sy.x=0.064; for other related statistics see Table 2]. (d) A seasonal comparison of L:W relationships. Large, sexually differentiated animals are heaviest in the fall and lightest in the winter. [LogW=a+bL+cL^2^; Summer: a=0.802, b=0.004, c=-2.012e-006, *n*=69, R^2^=1.00. Fall: a=1.081, b=0.003, c=-3.486e-007, *n*=15, R^2^=0.999. Winter: a=0.090, b=0.007, c=-3.927e-006, *n*=126, R^2^=0.995. Spring: a=0.745, b=0.005, c=-3.310e-006, *n*=179, R^2^=0.992. F_(9,901)_=3.903e+012; p<0.05*, curves are significantly different].

Figure 7A is a length histogram for the *E. cirrhatus* collected in this study. Figure 7B is a weight histogram, and Figure 7C shows the length:weight relationship for *n*=393 in a plot of weight vs length fitted to a second order polynomial equation. The median of the population was larger than the mean, which suggests that some very small individuals in the sample shifted the mean downward. 

Comparisons among seasons revealed statistically significant differences among the L/W curves [F_(9,901)_=3.903e+012; p<0.05; Figure 7D]. Hagfish collected in the winter showed the least increase in weight with increasing TL; the greatest weight increase was seen in summer and fall. The trend suggests that food availability may not be uniform throughout the year and that this affects the condition of the animals. The lower weights for smaller individuals in the spring sample may be due to lingering effects of reduced winter food availability on new recruits to the population; indeed, larger individuals in the winter sample weighed less than those in spring, summer and fall. Intermediate-sized individuals at 500-550 mm tended to cluster near the same weight of around Log W=2.5 in all seasons as shown by inspection of Figure 7D (which corresponds to a weight of 300-375 g).

**Figure 8:**
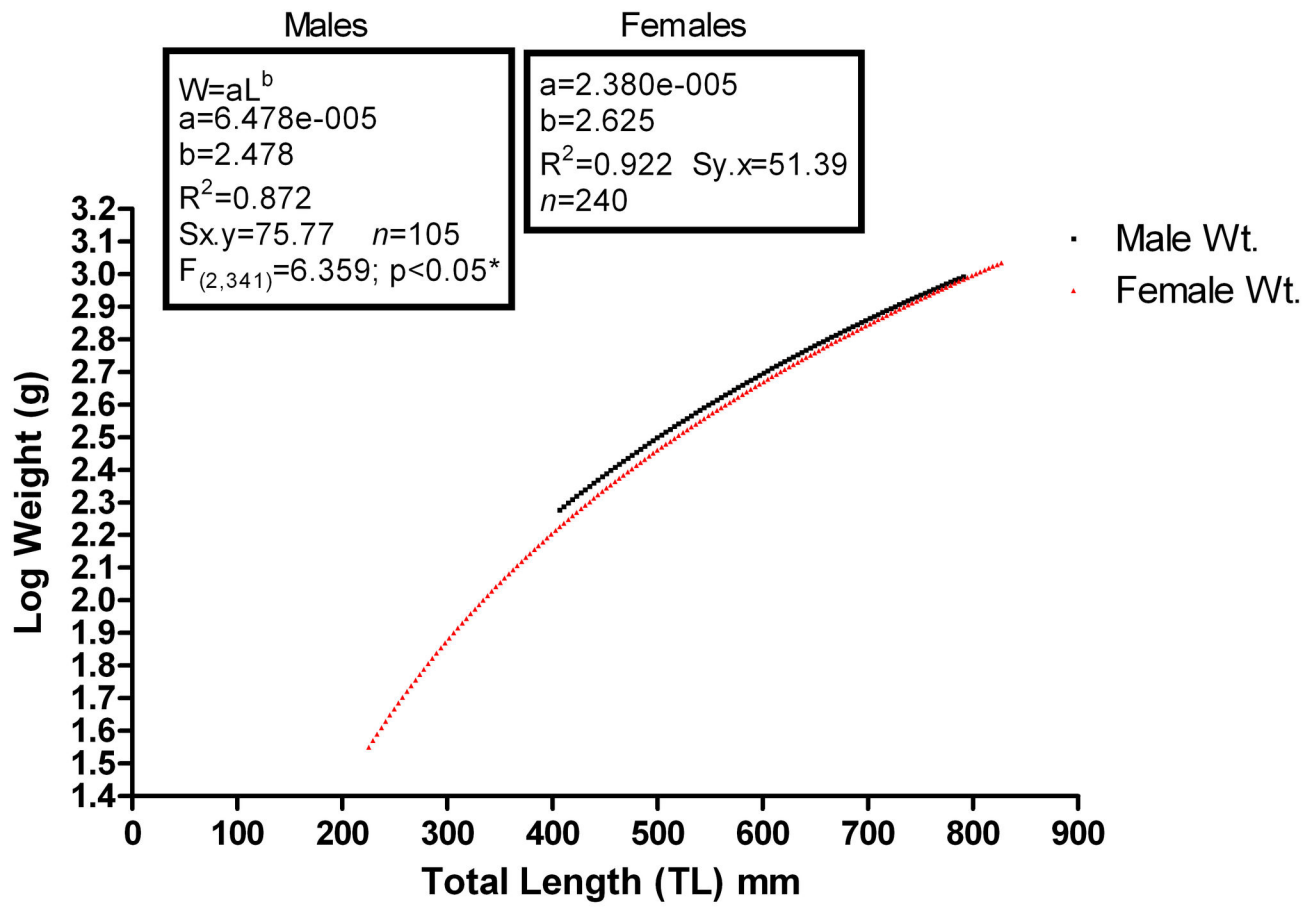
A comparison of male and female L:W curves. The curves are significantly different but parallel, with males heavier than females at all TLs.

In a comparison of male and female length:weight relationships with regard to possible differences in condition between the sexes, we fitted the data to the standard fisheries equation as shown in Figure 8. There was a significant difference between the length:weight relationships of males versus females [F_(2,341)_=6.359; p<0.05] with males slightly heavier than females at a given length, but the curves were parallel throughout the range of values. 

**Figure 9:**
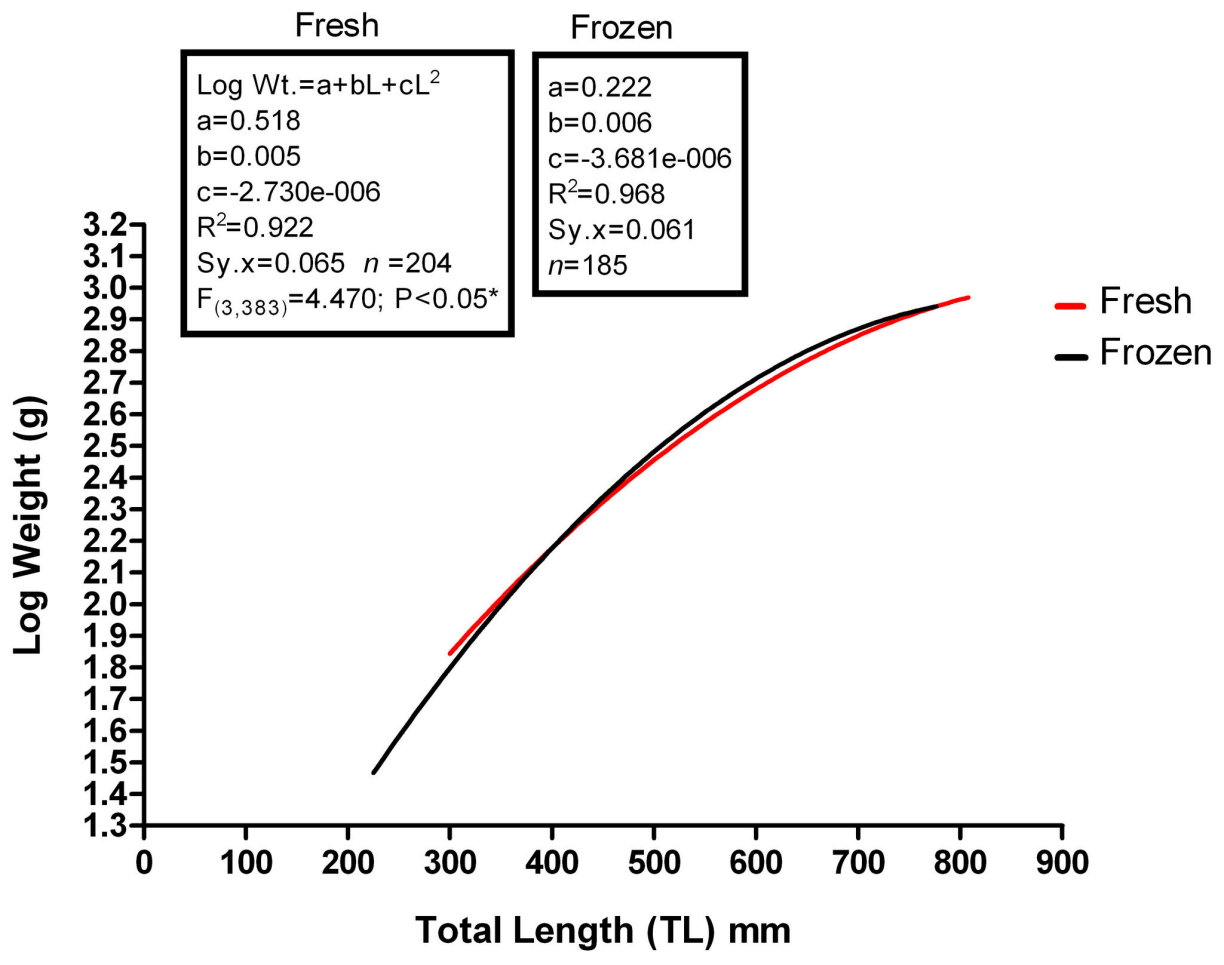
A comparison of fresh and frozen L:W curves. The curves are significantly different, with frozen specimens weighing slightly less at a given TL.

We compared the L/W data for fresh and frozen specimens fitted to a second order polynomial equation (Figure 9). The curves differed in two of the three equation parameters (a and b) [F_(3,383)_=4.470; p<0.05] with frozen specimens showing a better fit to the curve than the fresh specimens and with smaller standard deviation of weight. Frozen specimens tended to get slightly heavier at longer lengths but the shapes of the curves were very similar. 

 The assessment of the effect of preservation in formalin as shown by the matched t-test on the weights and the lengths before and after preservation revealed no statistically significant differences in weight after preservation (t=0.496; p>0.05) but there was a significant decrease in length. On average, preserved individuals were 8.75 percent shorter than fresh individuals in our small sample (t=11.46; p<0.05; *n*=24). 

### Reproductive status

**Figure 10:**
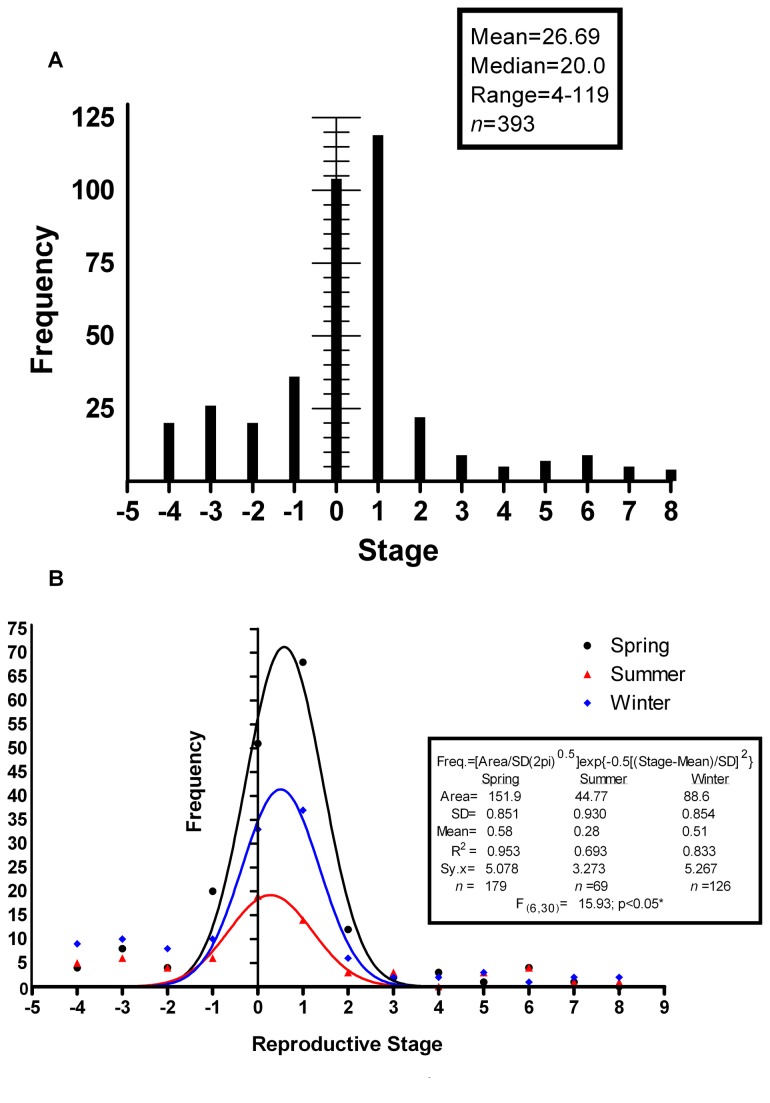
Reproductive status and seasonal patterns. (a) A frequency histogram for the reproductive stages introduced in Table 1. The majority of animals sampled in this study were either sexually undifferentiated or in the earliest stages of female gonadal development. (b) A seasonal comparison of the distribution of reproductive stages. The general pattern of distribution is similar in all seasons. Spring differs from other seasons in area but Spring, Summer, and Winter do not differ in Mean and SD.

Figure 10A presents a frequency histogram of the stages of gonadal development introduced in Table 1. Figure 10B shows the distribution of reproductive stages in Winter, Spring, and Summer; the *n* in Fall was too low for analysis. The form of the distribution was similar in these seasons, as shown by fitting the data to the Gaussian equation: Freq.=[Area/SD(2pi) ^0.5^]exp{-0.5[(Stage-Mean)/SD]^2^} yielding similar means and standard deviations (F_(4,30)_=0.440; p>0.05) but with different areas under the curves (F_(9,40)_=28.18; p<0.05). Differences in maximum height of curve (and hence area) reflect different numbers of individuals near the mean value of the sample from each season. As the form of all curves was Gaussian and the means and standard deviations were not different, this suggests that the average reproductive stage of the population did not change with season and that most individuals were females in Stage +1 or indeterminates in Stage 0. There was no indication of seasonal shifts associated with cyclical or seasonal spawning. Females with large eggs (32-35 mm in length), mature males, and postovulatory females were collected in all seasons. 

**Figure 11:**
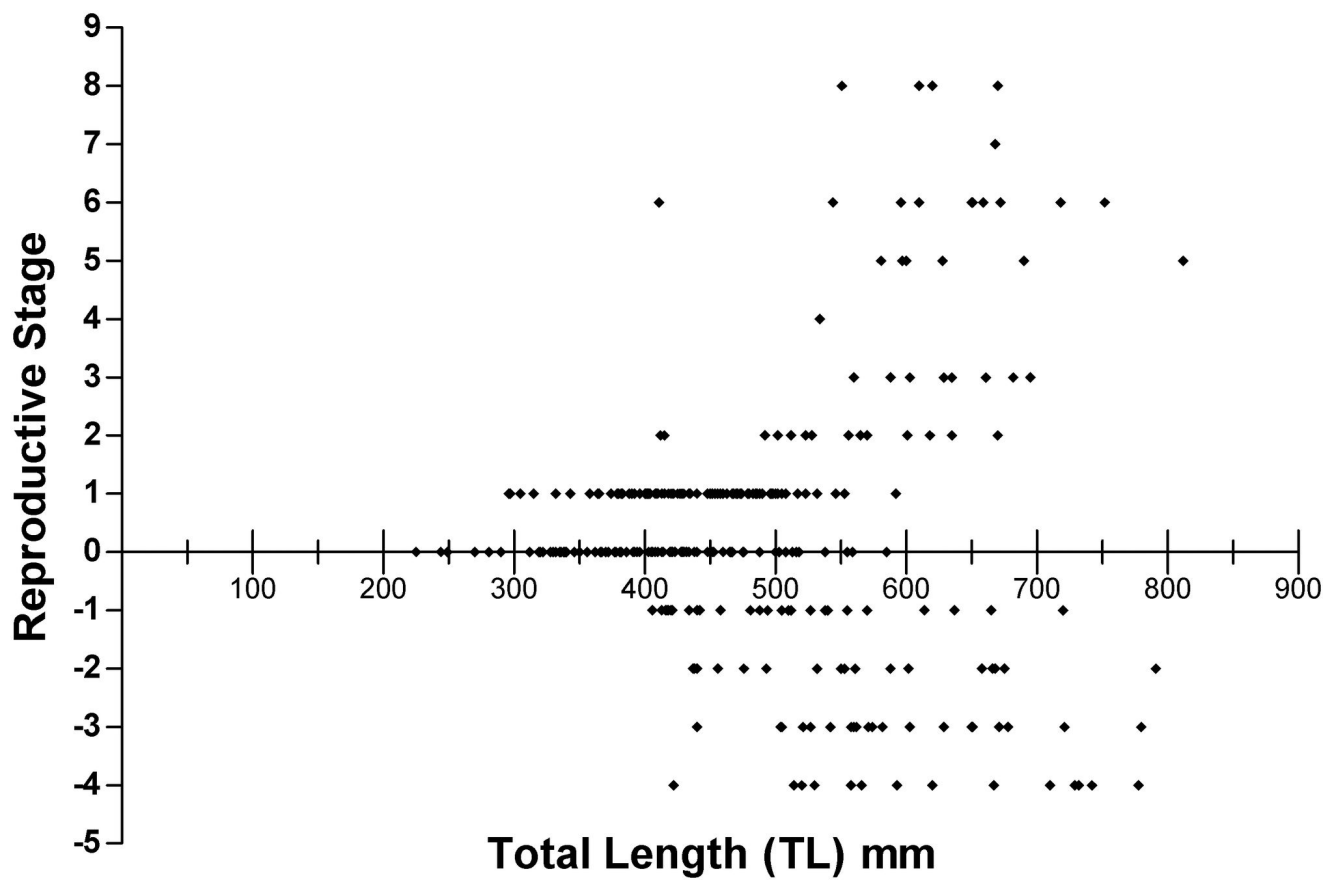
A scatterplot of reproductive stage vs total length. Note that all animals above 585 mm TL were readily identifiable as either male or female.

Figure 11A is a scatterplot of reproductive staging as a function of total length (TL). In our study, females were longer than 296 mm and males were longer than 406 mm. This suggests that males may begin gonadal development at a significantly greater TL than females. To test this, we subjected the TL at the onset of gonadal development of males vs females to the Mann-Whitney U test as there were substantially fewer males than females, which precluded a parametric test. The suggestion of delayed beginning of gonadal development in males is supported (Mann-Whitney U=792.5; p<0.05) but large specimens may be of either sex. The largest individual sampled was a female (812 mm TL) but the second largest was a male (791 mm TL).

Approximately 45% of the sampled population were females with developing eggs. The smallest female had a TL of 296 mm; the largest a TL of 812 mm. All females with a TL below 412 mm (n=28) had ovaries containing spherical eggs less than 1 mm in diameter and no evidence of prior spawning. The smallest female with eggs of 1-4 mm (Stage +2) had a TL of 412 mm; the smallest female with atrophied postovulatory follicles, often called corpora lutea or brown bodies, had a TL of 534 mm. The latter female had eggs of 15 mm in length, whereas females with large, flaccid postovulatory follicles had eggs less than 4 mm in length. This suggests that the first spawning cycle occurs at a TL above 412 mm but below 534 mm, a range that includes both the mean and the median TL of the sample population. 

**Figure 12:**
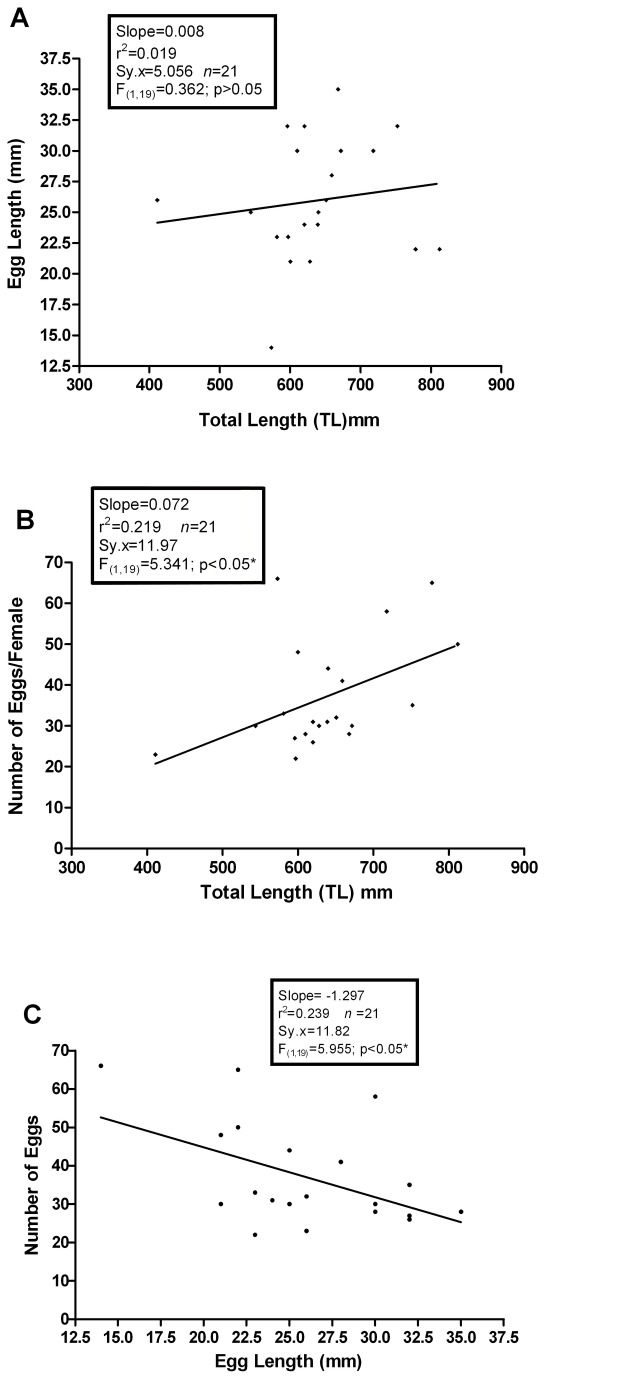
Female reproductive parameters. (a) Egg length as a function of TL. There is no signficant relationship here; larger females do not produce larger eggs than smaller females. (b) Egg number as a function of TL. Large female hagfish produce more eggs than smaller females. (c) A regression of the number of eggs as a function of egg length. For all females, the number of eggs under development decreases as the eggs grow larger.

There was no significant relationship between egg length and TL (F_(1,19)_=0.0.362; p>0.05; Figure 12A) but egg numbers and TL were positively related (F_(1,19)_=5.341; p<0.05: Figure 12B). Although larger females do not produce larger eggs they do produce a greater number of eggs. However, comparison of egg size with egg number indicates that the number of developing eggs decreases as the egg sizes increase (F_(1,19)_=5.955; p<0.05; Figure 12C).

None of the females examined contained shelled eggs ready to fertilize. Four specimens had large postovulatory follicles consistent with recent spawning activity and eggs of 1-4 mm , and another 7 had degenerating postovulatory follicles with developing eggs that ranged from 2-8 mm in length. The average number of near-mature (>32 mm) eggs or large, flaccid postovulatory follicles within a single female was 26 (n = 8, range: 20-35). We removed and weighed the 32 mm x 9 mm eggs from one female. At an average weight of 1.8 g, the 26 eggs comprised ~5% of the animal’s total weight. Only one specimen contained both testicular and ovarian tissues (an incidence of 0.25%). 

Males comprised 26% of the sample population. The smallest male had a TL of 406 mm and the largest a TL of 791 mm. The smallest “mature” male (Stage -4) had a TL of 422 mm. The most mature males observed had large, swollen testicular follicles and greatly distended cloacal glands. 

**Figure 13:**
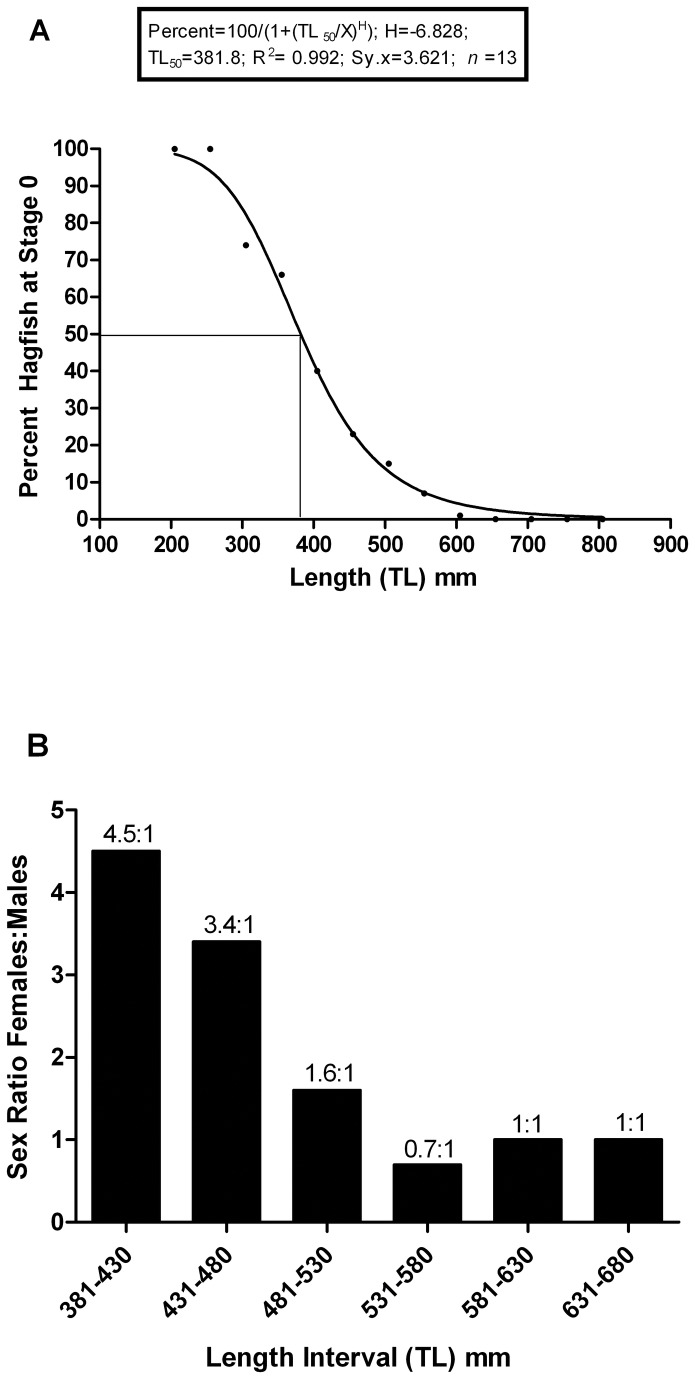
Gonadal development and sex ratios as a function of TL. (a) The percentage of hagfish at Stage 0 as a function of TL. Data were sorted into 50 mm TL intervals, and the percentage at Stage-0 determined as: (Stage-0s)/(females + males + Stage-0s) The percent values were plotted at the midpoint of each TL interval. (b) Sex ratios (female:male) as a function of TL. Data were sorted into 50 mm TL intervals. The sex ratio decreases from ~4.5:1 to 1:1 as the percentage of Stage 0 animals in the size interval decreases to zero (Figure 13a).

Approximately 28% of the *E. cirrhatus* sampled did not have macroscopically identifiable gonadal tissue (Stage 0). These animals ranged from 225-585 mm in total length. Although 74 animals greater than 585 mm were examined, all could be categorized as male or female. This suggests that by a TL of 585 mm, all members of the population have left Stage 0 and are undergoing gonadal maturation. Figure 13A indicates the percentage of Stage 0 individuals as a function of TL when the specimens are sorted into size groups at 50 mm length increments. The data were fitted to the three-parameter logistic equation, or Hill Equation, modified for X-axis as linear values and Y-axis normalised as percent and 100 defined as the Top: [Percent=100/(1+(TL_50_/X)^H^)] where X= Total length (mm) and H= Hill Slope]. Data points on the X-axis represented the midpoints of the 50 mm length intervals. The R^2^ value shows that 99.2% of the variance in gonadal development is accounted for by variance in length and the Sy.x (standard deviation of length) shows that data points are narrowly clustered near the curve. The TL_50_ for gonadal development in the sample population was 382 mm.

The sex ratio of females to males for our entire *E. cirrhatus* data-set was 1.8:1. However, when the data were sorted into size groups, we found that the sex ratio changed from 4.5:1 at 381-430 mm TL to 1:1 at >580 mm TL (Figure 13B). 

**Figure 14:**
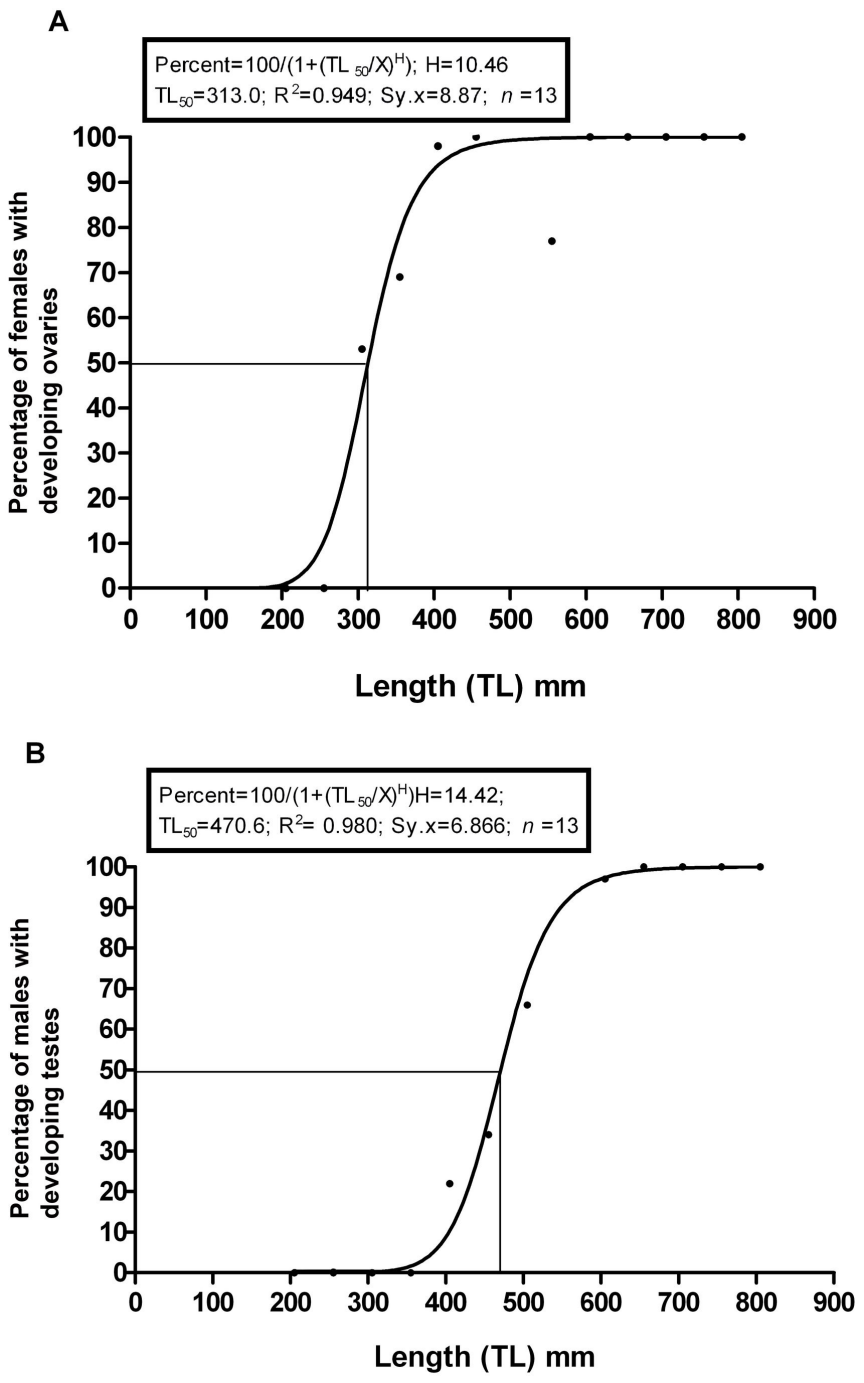
The initiation of female and male gonadal development. (a) The percentage of female hagfish with developing ovaries as a function of TL. The assumptions made are (1) that before male gonadal development has been initiated, and sex cannot be determined macroscopically (Stage 0), the genetic sex ratio is 1:1, and (2) the genetic sex ratio within each 50 mm TL intervals remains 1:1 throughout, although the individuals in a given interval may or may not have initiated gonadal development. (b) The percentage of male hagfish with developing testes as a function of TL. This graph is based on the same assumptions as part (a). Males begin gonadal development at a larger TL than females, and males at Stage 0 persist at TLs where all females have initiated ovarian development.

Because the sex ratio above 585 mm TL is 1:1, we would suggest that the genetic sex ratio of the population as a whole is 1:1 over all size classes, and that females predominate at small TLs because ovaries begin the process of growth toward maturation at a smaller TL than testes do in males. If one assumes that the genetic sex ratio is 1:1 regardless of whether or not morphological development has occurred, separate curves can be generated for females and males indicating the TL at which gonadal development becomes evident (Figures 14A and 14B). The TL_50_ for ovarian development in females is 313 mm, whereas the TL_50_ for testicular development in males is 471 mm. At the combined TL_50_ of 382 mm (Figure 13A), 90 percent of genetic females have developing ovaries, whereas only ~5 percent of males have developing testes. Note that these are not “maturation curves” or “maturity curves” [17] as the criterion is only that gonadal tissue, if present, can be identified as testes or ovaries. The time required to complete sexual maturation to the stage of fully shelled eggs or fully ripe testes is unknown. 

## Discussion

The morphological data-sets presented in Table 2 are in rough agreement with the work of other investigators, presented in Table 3 (*n*=53), although our data expanded the observed ranges of variation for several parameters. Where possible, data have been adjusted to be directly comparable; for example, measurements originally presented in thousandths of TL have been converted to percentages. 

**Table 3:** Morphometric data reported for *E. cirrhatus* in other studies.

Character	Mean	SD	Range	*n*
**Total length (mm)**				
Mincarone and Fernholm [18]			139-570	25
Mincarone and Stewart [19]			433-620	5
Fernholm [20]	720	73.5	560-830	10
**Weight (g)**				
Fernholm [20]	855	266	345-1250	10
**Prebranchial Length (%TL)**				
Mincarone and Fernholm [18]			23.6-27.6	25
Mincarone and Stewart [19]	22.2	7.8	21.4-23.9	5
Fernholm [20]	23	1.8	20.5-22.9	10
Strahan [21]			21-26	13
**Branchial Length (%TL)**				
Mincarone and Fernholm [18]			4.0-9.4	25
Fernholm [20]	8.1	0.52	7.2-8.7	10
Strahan [21]	7	1.1	6-9	13
**Trunk Length (%TL)**				
Mincarone and Fernholm [18]			47.3-56.8	25
Mincarone and Stewart [19]			52.5-56.8	5
Fernholm [20]	54.7	1.37	52.2-57.7	10
**Tail Length (%TL**)				
Mincarone and Fernholm [18]			13.3-17.7	25
Fernholm [20]			13.3-17.9	10
**Depth, trunk (%TL**)				
Mincarone and Fernholm [18]			6.9-10.2	25
Fernholm [20]	9.4	1.15	7.5-11.0	10
Strahan [21]	8.5	0.3	7-9	13
**Depth, cloaca (%TL)**				
Mincarone and Fernholm [18]			6.1-8.2	25
Mincarone and Stewart [19]			5.7-7.5	5
Fernholm [20]	7.2	0.66	6.1-8.5	10
**Depth, tail (%TL)**				
Mincarone and Stewart [19]			7.7-9.1	5
Fernholm [20]	8.7	1.01	7.0-10.0	10
**Total cusps**				
Mincarone and Fernholm [18]			44-51	25
Mincarone and Stewart [19]			43-51	5
Fernholm [20]	47	1.3	44-48	10
**Multicusps, outer/inner**				
Mincarone and Fernholm [18]	3/3			25
Mincarone and Stewart [19]	3/3			5
Fernholm [20]	3/3			10
Strahan [21]	3/3			13
**Unicusps, outer/inner**				
Mincarone and Fernholm [18]			8-10/8-10	48
Mincarone and Stewart [19]			8-11/NR	5
Fernholm [20]			8-9/8-9	10
Strahan [21]			6-9/6-9	13
**Total slime pores (left side)**				
Mincarone and Fernholm [18]			79-87	26
Fernholm [20]	88	1.5	83-90	10
Strahan [21]	87	2.7	86-90	13
**Prebranchial slime pores**				
Mincarone and Fernholm [18]			16-20	26
Mincarone and Stewart [19]			16-20	5
Fernholm [20]	16	0.8	15-17	10
Strahan [21]	16	1.0	15-18	13
**Branchial slime pores**				
Mincarone and Fernholm [18]			5-7	26
Fernholm [20]	7	0	7-7	10
Strahan [21]	6.5	0.2	6-7	13
**Trunk slime pores**				
Mincarone and Fernholm [18]			45-51	26
Fernholm [20]	52	1.8	50-57	10
Strahan [21]	51	1.7	49-54	13
**Tail slime pores**				
Mincarone and Fernholm [18]			10-14	26
Fernholm [20]	13.3	0.7	12-14	10
Strahan [21]	13	1.0	12-14	13

As has been suggested for *Myxine glutinosa* [3], juvenile *E. cirrhatus* may target different feeding resources than older animals, and not be attracted to carrion-baited traps. However, Mincarone and Fernholm [18] reported specimens of shorter TL than our study. Two factors may be responsible for this difference:

1. The presence of 17 mm escape holes in the commercial traps may have allowed small individuals to leave the trap during hauling. Although many of the animals caught are smaller in diameter than the escape holes, those small animals are probably trapped in the bait, caught in the slime within the trap, or tangled with larger specimens. 

2. The leather and meat fisheries cannot utilize very small hagfish, and when time and sea conditions permit, the crew discarded them. 

There are relatively few available data concerning the lengths and weights of hagfish targeted by fisheries operations. Our data indicate that the hagfish landed by the *E. cirrhatus* fishery in New Zealand had a mean length of 486 mm and a median length of 468 mm. Based on the reproductive status of the samples (Figures 9A and 10), we would classify this fishery as targeting immature animals. Kuenstner [22] and Grant et al. [23] reported on experimental fisheries for *M. glutinosa* in the western North Atlantic. Trawl surveys indicated that the fisheries catches reflected the population as a whole [24] with an average TL between 405 mm (spring) and 426 mm (fall). *M. glutinosa* within the Gulf of Maine are sexually indeterminate (Stage 0) below 400 mm TL [4], whereas fisheries sampling off Newfoundland, where *M. glutinosa* matures at a smaller size, indicated a gonadal development (“maturation”) TL_50_ of ~392 mm [23]. Thus the New England *M. glutinosa* fishery, like the New Zealand *E. cirrhatus* fishery, is taking large numbers of juvenile animals. Barss [25] provided catch record information from *E. stoutii* targeted by fishermen off the Pacific coast of the U.S. that can be correlated with the biological information for this species from Johnson [26]. The mean hagfish TL for fishery samples over 1988-1989 was 396 mm, whereas the biological data indicated the average size for females with large developing eggs (>15 mm) was 376.03 +/- 31.35. Thus it would appear that all three of these hagfisheries (*M. glutinosa, E. stoutii*, and *E. cirrhatus*) remove large numbers of juveniles from the targeted populations. 

In New Zealand, the depth zone of 400-500m was selected by trial and error as the fishermen sought to maximize their catch. The size distribution at other depths may be quite different. For example, Johnson [26] found that small *E. stoutii* were at peak abundance at 250m, with large males more common at 100m, and large females more common at 100m, although specimens of all sizes and sexes were collected throughout the depth range. Thus a collection made at 100m would potentially yield a different size distribution and sex ratio from a collection made at 250m or 500m. It is possible that the maximum trap yield of marketable hagfish is found within a depth zone where the preferred ranges of males, females, and juveniles overlap, but this cannot be determined without extensive transect surveys. 

Linkage between testicular maturation and cloacal gland development was reported by Tsuneki et al. [27] for *E. burgeri*. It is assumed that these enlarged glands play some role in spawning, but details of cloacal gland function are unknown since spawning in hagfishes has not yet been observed. Tsuneki et al. [27] reported that enlarged cloacal glands were characteristic of both mature males and mature females with large eggs, but we observed only one female with unusually large cloacal glands. This could imply that the female cloacal glands have different functions in *E. burgeri* vs *E. cirrhatus*. However, since none of the females collected in our study contained fully developed, shelled eggs, it is possible that female cloacal glands in *E. cirrhatus* enlarge immediately prior to spawning. 

Gorbman [28] reported that female *E. stoutii* were longer than 200 mm in total length (TL) and males are longer than 280 mm TL; the largest animals in his study were usually females. Our data suggest that in *E. cirrhatus*, as in *E. stoutii*, female sexual maturation is initiated at a smaller size, but that large individuals of *E. cirrhatus* may be either male or female. We reported the incidence of hermaphroditism to be 0.25% in *E. cirrhatus*; the incidence of hermaphroditism in *E. stoutii* over 230 mm TL is comparably low (0.3%; 28), and Patzner [29] estimated the rate of hermaphroditism in *E. burgeri* at <0.1%.

A correlation between the number of large eggs and female TL has been reported for *E. burgeri* [30], the only known species with a synchronous spawning cycle [29,31,32,33]. Similarly, we found a correlation between egg numbers and female TL in *E. cirrhatus*. Tsuneki et al. [30] and Nozaki et al. [34] found a clear seasonal pattern in size frequencies and reproductive state, with increases in egg size and testicular development evident in their population samples. These patterns, which would have been apparent in sampling conducted at 3-month intervals, were not seen in our study although we did detect seasonal variations in the length-weight relationships. Our findings are thus consistent with the contention that *E. cirrhatus*, like most other hagfishes studied, has no specific breeding season. However, Powell et al. [35] suggested that seasonality may exist even in the absence of patterns of gonadal development, based on changes in gonadal hormone levels; that hypothesis has not as yet been confirmed. 

Throughout the year our collection contained relatively few very large adults and no gravid females. We cannot determine whether this is because gravid females do not feed or because spawning activities occur elsewhere, as suggested by Patzner [33] who noted the paucity of gravid females in the data from other species. 

The presence of large numbers of “sterile” individuals has been reported for populations of *E. stoutii* in the Pacific [26] and for *M. glutinosa* in the North Atlantic [4,36,37]. Sterile animals include animals without any evidence of gonadal tissue and animals with abnormal or degenerating ovarian and testicular tissues [33]. In *M. glutinosa*, the incidence of sterility in animals over 400 mm TL has been reported to be 25% [4] in the Western Atlantic and 13% in the eastern Atlantic [35], with sterile individuals reported across the full size range of the species. It is possible that animals lacking gonads and classified as “sterile” in these studies were the equivalent of our Stage 0 animals, awaiting an as-yet unknown trigger to initiate gonadal development. 

 Gorbman [28], who noted that small *E. stoutii* were neuters or immature females, suggested that the species was protogynous. However, Johnson [26] noted that the sex ratio in *E. stoutii* gradually decreased from 1.8:1 at small sizes to roughly 1:1 for animals at or above 380 mm TL. This parallels our findings on *E. cirrhatus*, suggesting that the female bias at small sizes may be the result of asynchronous female:male morphological differentiation rather than juvenile protogyny. Similarly, the TL size distributions of each collection, potentially compounded by differences in the rates and timing of gonadal growth and development, may account for the varied sex ratios that have been reported among collections of other *Eptatretus* species. 

All data on hagfishes suggest that their reproductive potential is very low, and this species is no exception. Relevant factors in this report include (1) the presence of a significant number of undifferentiated animals over a broad size range (2), a low fecundity, with spawning females each releasing fewer than 30 eggs (3), the relatively small percentage of adult females that contain large eggs, and (4) the paucity of females with large, flaccid postovulatory follicles. Factors reported for other species but as yet undetermined for *E. cirrhatus* include the extended time period required to produce large megalecithal eggs for each spawning cycle and for the fertilized eggs to undergo embryonic development. The egg production period for asynchronous species may be 2-3 years [29]. Although the total duration of embryonic development prior to hatching has not been determined, development to the neurulation stage in *E. burgeri* required a minimum of 7 months [38]. These factors have obvious implications for the development of a sustainable fishery. 

Historically, hagfisheries worldwide have been characterized by overfishing, population declines, and catch reductions [24,39,40,41]. Yet from a regulatory perspective it is difficult to set politically viable guidelines for a fishery when the population size, total biomass, and distribution is unknown, and when individual growth rates, recruitment rates, and longevity remain to be determined. In view of the limited reproductive potential of hagfishes and the fact that hagfisheries tend to target juvenile animals, it would be prudent to maintain tight control over emerging or experimental hagfish fisheries, through permits, mandated escape hole sizes, and monitoring of catch rates and discards until additional biological and ecological data can be obtained. 

## Supporting Information

Figure S1 **(**a**) The 72m Korean fishing vessel ShinJi, berthed at Wynyard Wharf, Auckland.** (b) Hagfish traps are stored on the upper deck. Each 200 L barrel has a funnel shaped entry at the top and escape holes in the sides.
(c) A single freezer bag of hagfish, weighing approximately 25 kg and measuring 600mm x 300mm x 200mm.
(d) An open freezer bag of hagfish; as the contents thaw individual specimens are removed, weighed, measured, and sexed.
(TIF)
