# Supplementary figures and images for: Morphometics and Gonadal Development of the Hagfish Eptatretus cirrhatus in New Zealand
Source: PLoS One. 2013 Nov 8;8(11):e78740. doi: 10.1371/journal.pone.0078740 (PMC3826707; doi:10.1371/journal.pone.0078740)

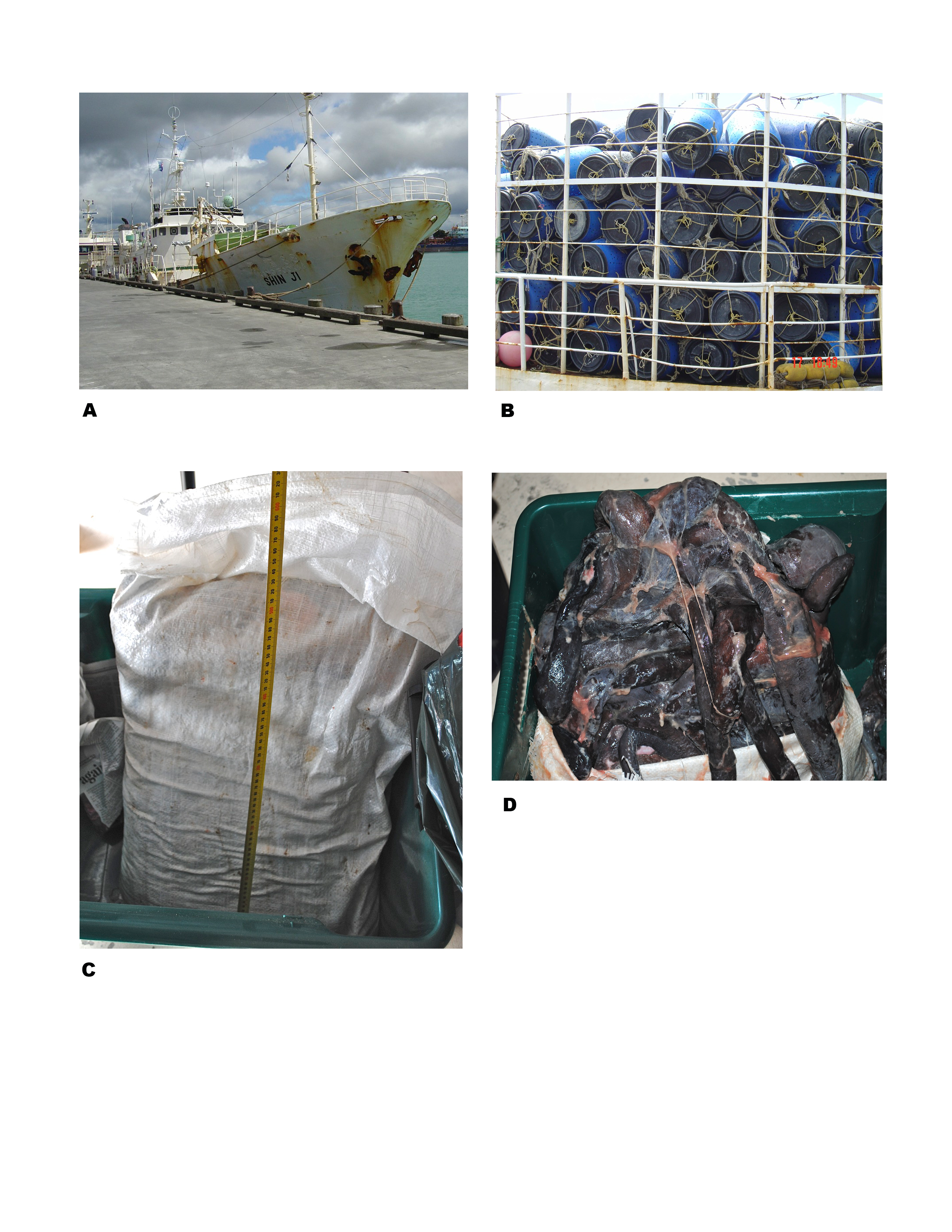

Supplement: Figure S1 — (a) The 72m Korean fishing vessel ShinJi, berthed at Wynyard Wharf, Auckland. (b) Hagfish traps are stored on the upper deck. Each 200 L barrel has a funnel shaped entry at the top and escape holes in the sides. (c) A single freezer bag of hagfish, weighing approximately 25 kg and measuring 600mm x 300mm x 200mm. (d) An open freezer bag of hagfish; as the contents thaw individual specimens are removed, weighed, measured, and sexed. (TIF) [file pone.0078740.s001.tif]
